# Documentation of vaccine handling and service delivery at outreach immunization sessions across 27 districts of India

**DOI:** 10.1016/j.heliyon.2018.e01059

**Published:** 2018-12-18

**Authors:** Manoja Kumar Das, Narendra Kumar Arora, Thomas Mathew, Bhadresh Vyas, Monica Sindhu, Abhishek Yadav

**Affiliations:** a The INCLEN Trust International, F1/5, Okhla Industrial Area, New Delhi, 110020, India; b Department of Community Medicine, Government Medical College, Thiruvananthapuram, Kerala, 695011, India; c Department of Pediatrics, M P Shah Medical College, Jamnagar, 361008, Gujarat, India

**Keywords:** Public health, Vaccines

## Abstract

**Background:**

Outreach sessions constitute a major share of routine immunization service under national program in India.

**Objective:**

To document the organisation, logistics, vaccine handling and services delivered during outreach sessions in India.

**Method:**

This cross-sectional study was undertaken at 136 outreach sessions across 27 districts in three states (Bihar-62, Gujarat-43 and Kerala-31). Data was collected on session organization, vaccine supply, handling, beneficiary interaction, documentation, and waste handling.

**Results:**

All essential items and vaccines were available at 52.2% and 59.7% of sessions. The overall beneficiary turnout was 72.6%. Matching diluents were available for 94.4% of lyophilised vaccine vials. All four messages were given to 58.8% beneficiaries and 40% were advised to wait for 30 minutes. Few sites received vaccine vials with unusable vaccine vial monitors and frozen free-sensitive vaccine vials.

**Conclusion:**

Program attention is needed to improve organisation, logistics and vaccine handling at the outreach sessions to ensure optimal service delivery and beneficiary experience. The supportive supervision and monitoring must be strengthened focusing on updated beneficiary list, vaccine handling, counselling and waste handling.

## 1 Introduction

Vaccination is one of the most effective and powerful preventive measures for reducing the deadly morbidities and thereby mortality. India has expanded the antigens envelope under the universal immunization program (UIP) in last few years to eight vaccines universally and four vaccines (rotavirus, pneumococcus, rubella and Japanese encephalitis) in select states. Following National Technical Advisory Group on Immunization recommendations, rotavirus and pneumococcus vaccines are being scaled up nationally in a phased manner. UIP in India covers about 30 million pregnant women, 26 million infants born annually and about 100 million 1–5 year children [1]. These beneficiaries are vaccinated through over 9 million immunization sessions (fixed, outreach and mobile), and the outreach sessions constitute a major share (59% of the vaccination given through the public health system) [1, 2]. The immunization sessions are usually linked to the Village Health and Nutrition Days (VHNDs) integrating the health and nutrition outreach services. According to the National Family health Survey-4 (2015–16), the full immunization coverage of children aged 12–23 months was 63.9%, reflecting a small rise over last decade [3]. Also the private share of immunization increased from 7.2% to 16.7% over the last decade [3]. Concurrent monitoring under the routine immunization programme indicated operational gaps as the reason for missing vaccination in 10% of cases [4]. The Auxiliary Nurse Midwives (ANM), Accredited Social Health Activists (ASHA) and Anganwadi Workers (AWW) coordinate for effective conduct of the outreach sessions. Apart from the vaccination, vaccine handling, delivery of four key messages, counselling and documentation are also important part of the session. The ANM is expected to remind parents about the four key messages including (a) what vaccine was given and what disease it prevents, (b) when and where to come for the next visit, (c) what are the minor side effects and how to deal with them and (d) safe keep of the immunization card. These are part of the supervision and monitoring of the outreach sessions under UIP. The handbooks for doctors and health workers on routine immunization provides guidelines for microplan, preparation, conduct of outreach session, vaccine handling and related reporting [1, 5]. There are patchy and limited information available about the logistic availability, delivery of counselling and the key messages to the beneficiaries during outreach sessions. To improve access and quality of the outreach sessions, there is need to document the vaccine handling and service delivery in different contexts.

This study was undertaken to document the logistic readiness, vaccine handling and services delivered during outreach sessions in three states of India.

## 2 Methods

**Fig. 1:**
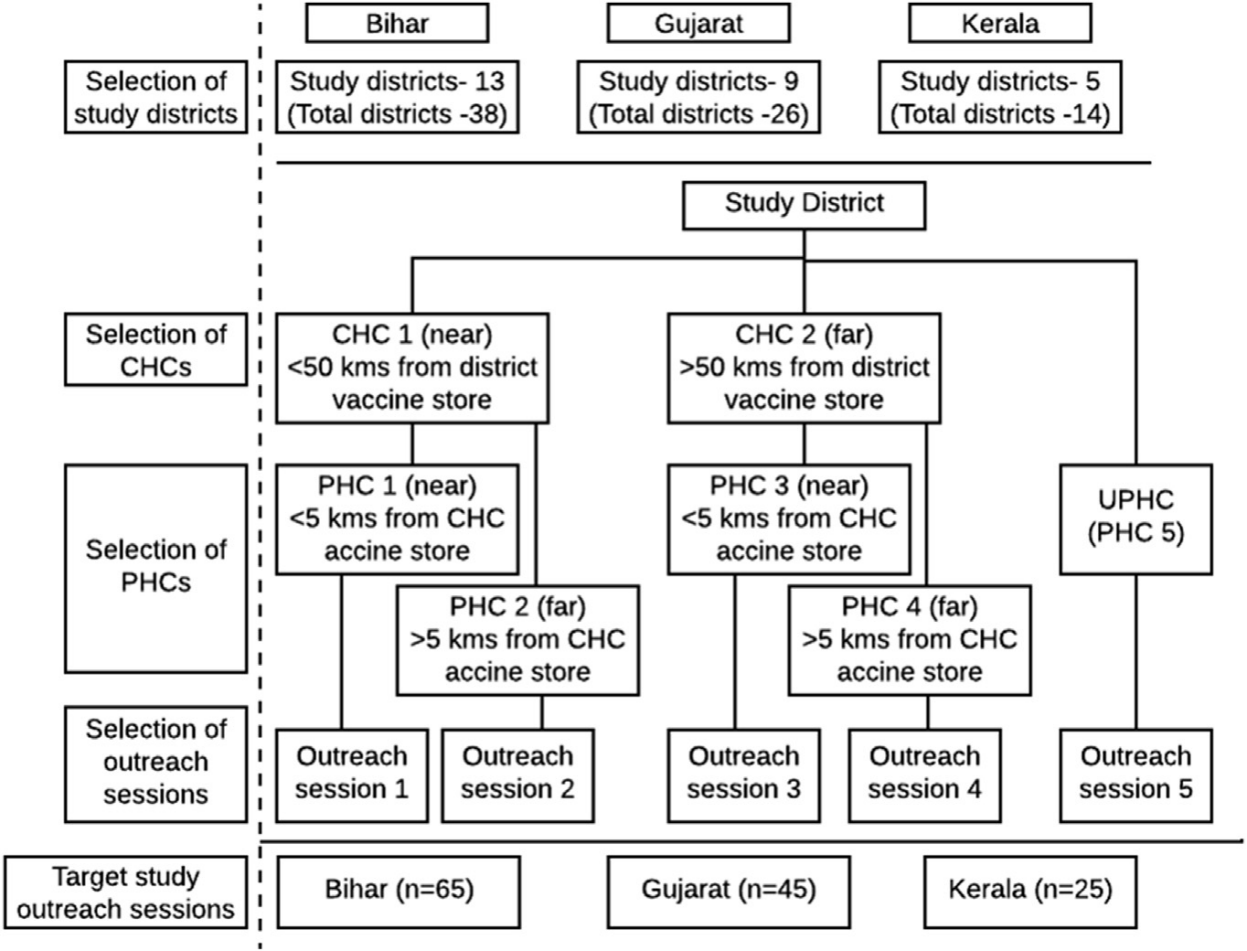
Process of selection of outreach sessions in the study districts. *Note: CHC: Community health centre; PHC: Primary health centre, UPHC: Urban primary health centre*.

This cross-sectional study was conducted in three states (Bihar, Gujarat and Kerala) during 2014–15. The states selected represented three different levels of routine immunization coverage (lowest, Bihar 49%, moderate, Gujarat 56.6% and highest, Kerala 81.5% strata in India according to Coverage Evaluation Survey 2009), three different governance zones and geography. In each state, one third of the districts were covered under the study. A total of 27 districts, 13 in Bihar (total districts-38), 9 in Gujarat (total districts-26) and 5 in Kerala (total districts-14) were randomly selected. In each study district, five outreach sessions were selected through a multistage approach based on the distance from the district vaccine store, as shown in Fig. 1.

Thus a total of 135 outreach sessions (Bihar- 65; Gujarat- 45 and Kerala - 25) from the 27 study districts were selected for observation. Two members observed the complete outreach sessions and interacted with the ANM using structured tools. Data on session organization, vaccine supply, vaccine handling, beneficiary interaction, documentation, and waste handling were collected. Double data entry was done and the entries were matched for correctness. Data was analysed using Microsoft Excel and STATA version 12 (StataCorp, LLC, Texas, USA). This being a descriptive study, the actual numbers and proportions of the study variables have been reported. Statistical tests for significance have not been applied. Administrative approvals from national and state authorities and ethical approval from the INCLEN institute ethics committee (protocol no IIEC-006) were obtained.

## 3 Results

A total of 136 outreach sessions in three states (Bihar-62, Gujarat-43 and Kerala-31) were observed. In five districts (Bihar-3 and Gujarat-2), only four outreach sessions could be observed and six additional sessions in Kerala were included. The average population covered by the outreach sessions was 3374 (Bihar-4129, range 1035–13140; Gujarat-1553, range 358–3814 and Kerala-4441, range 1045–13548).

As shown in Table 1, most of sessions were held as per the schedule and site plan. Only 7.4% of the sessions in Kerala were linked with the VHNDs compared 71% and 62.8% in Bihar and Gujarat, respectively. Overall, the ASHAs and AWWs were present in 75.7% and 76.5% of the sessions. The availability of various essential items for vaccination, waste segregation and recording were variable across the states (Table 1). All the essential items were available at 71 (52.2%) sessions (Bihar-32.3%; Gujarat-62.7% and Kerala-61.3%).

**Table 1:** Organisation of the sessions and availability of the items.

	State			Total (n = 136) n (%)
	Bihar (N = 62) n (%)	Gujarat (N = 43) n (%)	Kerala (N = 31) n (%)	
*A. Session organisation*				
A.1 Scheduled session	60 (96.8)	43 (100)	29 (93.5)	132 (97.1)
A.2 Fixed site for session	61 (98.4)	42 (97.7)	27 (87.1)	130 (95.6)
A.3 Sessions linked with VHND	44 (71)	27 (62.8)	2 (6.5)	73 (53.7)
A.4 ASHA present	44 (71%)	35 (81.4)	24 (77.4)	103 (75.7)
A.5 AWW present	53 (85.5%)	33 (76.7)	18 (58.1)	104 (76.5)
*B. Essential items/supplies*				
B.1 Cotton swabs	59 (95.2)	42 (97.7)	30 (96.8)	131 (96.3)
B.2 Auto-disable syringes	62 (100)	43 (100)	30 (96.8)	135 (99.3)
B.3 Disposable syringes	59 (95.2)	40 (93)	22 (71)	121 (89)
B.4 Clean water for swabs	40 (64.5)	39 (90.7)	30 (96.8)	109 (80.1)
B.5 Water & soap for hand wash	44 (71)	34 (79.1)	29 (93.5)	107 (78.7)
B.6 Hub cutter∗	38 (61.3)	41 (95.3)	21 (67.7)	100 (73.5)
B.7 Waste collection containers	28 (45.2)	40 (93)	30 (96.8)	98 (72.1)
B.8 Blank immunization cards	57 (91.9)	36 (83.7)	19 (61.3)	112 (82.4)
B.9 Tally sheets of beneficiaries	59 (95.2)	29 (67.4)	20 (64.5)	108 (79.4)
B.10 Vaccine record/registers	59 (95.2)	41 (95.3)	30 (96.8)	130 (95.6)
B.11 Medicine (Paracetamol)	62 (100)	43 (100)	31 (100)	136 (100)
B.12 All desired items available	25 (32.3)	27 (62.7)	19 (61.3)	71 (52.2)

Note: VHND- Village Health and Nutrition Day.

∗ Hub cutter used to cut the hubs of the syringes after use at the session site itself and collect the sharps (needles) in the hub cutter container.

Table 2 shows the vaccine logistics and supply status for the sessions in these districts. Many of the vaccine carriers in Kerala were packed by ANMs and carried to the session sites themselves. At least one vial of each vaccine was available at 59.7% sessions (Bihar-79%, Gujarat-74.4%, and Kerala-25.8%). Mismatches in BCG and measles vaccine and diluents vials (numbers) were observed at several sites. At few sites in Bihar, icepacks contained only cold water at the time of receipt. At two sites in Kerala the labels on three vaccine vials were missing. At three sites, four vaccine vials had unusable vaccine vial monitor (VVM). At three sites, eleven freeze-sensitive vaccine vials were found frozen.

**Table 2:** Vaccine logistics and availability at session sites.

Parameters	Bihar (n = 62) n (%)	Gujarat (n = 43) n (%)	Kerala (n = 31) n (%)	Total (n = 136) n (%)
*A. Vaccine carrier packed by*				
A.1 Cold chain handler	51 (82.3)	27 (62.8)	8 (25.8)	86 (63.2)
A.2 ANM herself	6 (9.7)	13 (30.2)	22 (71)	41 (30.1)
*B. Vaccine carrier transport*				
B.1 Motorised transport	16 (25.8)	40 (93)	31 (100)	86 (63.2)
B.2 Non-motorised transport	43 (69.4)	3 (7)	0 (0)	46 (33.8)
B.3 By foot/walking	3 (4.8)	0 (0)	0 (0)	3 (2.2)
*C. At least one vial/ampoule of vaccines available*				
C.1 BCG	49 (79)	32 (74.4)	9 (29)	90 (66.2)
C.2 DPT	57 (91.9)	43 (100)	28 (90.3)	128 (94.1)
C.3 OPV	48 (77.4)	43 (100)	31 (100)	122 (89.7)
C.4 Measles	61 (98.4)	41 (95.3)	29 (93.5)	131 (96.3)
C.5 TT	56 (90.3)	43 (100)	29 (93.5)	128 (94.1)
C.6 HBV	50 (80.6)	32 (74.4)	8 (25.8)	90 (66.2)
C.7 Pentavalent∗	NA	43 (100)	31 (100)	74 (100)∗
*D. Matching vaccine and diluent available*				
D.1 BCG@	46 (93.9)	30 (93.8)	6 (66.7)	81 (91.1)
D.2 Measles#	59 (96.7)	40 (97.6)	29 (100)	128 (97.7)
*E. Status of icepacks at receipt*				
E.1 Fully frozen	29 (46.7)	27 (62.8)	24 (77.4)	80 (58.8)
E.2 Partial frozen	29 (46.7)	16 (37.2)	7 (22.6)	52 (38.2)
E.3 Cold water only	4 (6.4)	0 (0)	0 (0)	4 (3)

Note: * Pentavalent vaccine was not introduced into immuization program schedule in Bihar at the time of data collection. The proportion for denominator included only Gujarat and Kerala.

@ The proportions calculated using the number of sessions with available BCG vaccine vials as the denominator indicated in row C.1.

# The proportions calculated using the number of sessions with available Measles vaccine vials as the denominator indicated in row C.4.

Table 3 summarises the vaccine handling at the session sites. About half of the vaccinators washed hands before vaccination. At 16 (11.8%) sites freeze-sensitive vaccines were placed in contact with the icepacks during sessions. At majority (89.7%) of the session sites, the time of reconstitution for BCG/Measles vaccines were written and 81.6% of the reconstituted vials were used within 4 hours. The used BCG/Measles vials were returned to store from 73.5% sessions, while either discarded at the session site or segregated with waste at the other sites. Partially and completely used vials under open vial policy were returned to store from 87.5% session sites. After vaccination, 63.7% of the auto-disable and 50.7% of the disposable syringes were handled appropriately.

**Table 3:** Vaccine and syringes handling practices at the sessions.

	State			
	Bihar (n = 62) n (%)	Gujarat (n = 43) n (%)	Kerala (n = 31) n (%)	Total (n = 136) n (%)
*A. Checking on receipt*				
A.1 Checked the vaccine/diluents on receipt	58 (93.5)	42 (97.7)	28 (90.3)	128 (94.1)
A.2 Checked ice pack status	54 (87.1)	27 (62.8)	25 (80.6)	106 (77.9)
A.3 Hand washed before vaccination	29 (46.8)	23 (53.5)	21 (67.7)	73 (53.7)
*B. Vaccine vials correctly placed during session*				
B.1 Non-freeze sensitive vaccines∗	62 (100)	43 (100)	31 (100)	136 (100)
B.2 Freeze sensitive vaccines#	53 (85.5)	39 (90.7)	28 (90.3)	120 (88.2)
B.3 Reconstituted vaccines@	60 (96.8)	43 (100)	29 (93.5)	132 (97.1)
*C. BCG/Measles vials handling*				
C.1 Time of reconstitution written	55 (88.7)	40 (93)	27 (87.1)	122 (89.7)
C.2 Used within 4 hours	49 (79)	38 (88.4)	24 (77.4)	111 (81.6)
*D. Used vials (not under open vial policy)*@				
D.1 Discarded at site	5 (8.1)	3 (7)	3 (9.7)	11 (8.1)
D.2 Returned back to store	54 (87.1)	28 (65.1)	18 (58.1)	100 (73.5)
D.3 Segregated with waste	3 (4.8)	12 (27.9)	10 (32.2)	25 (18.4)
E. Partially/completely used vials (under open vial policy)*#				
E.1 Discarded at site	3 (4.8)	2 (4.7)	2 (6.5)	7 (5.1)
E.2 Returned back to store	56 (90.3)	37 (86)	26 (83.9)	119 (87.5)
E.3 Segregated with waste	3 (4.8)	4 (9.3)	3 (9.7)	10 (7.4)
*F. Correct syringe/needle handling after use*				
F.1 Auto-disable syringes	32 (51.6)	39 (90.7)	15 (48.4)	86 (63.2)
F.2 Disposable syringes/needles	28 (45.2)	33 (76.7)	8 (25.8)	69 (50.7)

Note:

∗ Non-freeze sensitive vaccines following open vial policy- oral polio vaccine (OPV).

# Freeze sensitive vaccines- Diptheria-pertussis-tetanus toxoid (DPT); pentavalent vaccine (Diptheria-Pertussis-Tetanus toxoid-Hemophilus influnzae b, Hepatitis B); Tetanus toxoid; and Hepatitis B vaccine.

@ Reconstituted vaccines/vaccines not under open vial policy- BCG and Measles vaccine.

∗# Open vial policy allows use of reuse of partially used multidose liquid vaccine vials in subsequent session(s) up to four weeks subject to appropriate vaccine handling and temperature stability conditions are met to reduce vaccine wastage. Open vial policy is applicable to DPT, tetanus toxoid, Hepatitis B, pentavalent, oral polio, inactivated polio vaccines, and pneumococcal vaccines.

The overall turnout was 72.6% for the outreach sessions (Bihar-103%, Kerala-62.6% and Gujarat-66.8%). Turnout of the children was high in Bihar (84.7%) followed by Kerala (71.2%) and Gujarat (64.9%). Turnout of the pregnant women was also higher in Bihar (122%) compared to Gujarat (68.7%) and Kerala (54.1%). In 27 sessions (Bihar-15, Gujarat-9, Kerala-3), at least one of the supplied vaccine was finished. Due to unavailable vaccines, in 15 sessions (Bihar-6, Gujarat-7 and Kerala-2), some beneficiaries were returned without vaccination.

As reflected in Table 4, the vaccinators delivered all four key messages to the beneficiaries in 58.8% (56.5%–64.5%) of the sessions. Explanation before vaccination was the commonest missed component across all states. About 40% of the beneficiaries were advised to wait for 30 minutes. Paracetamol was disbursed to 85.3% of the beneficiaries.

**Table 4:** Vaccination and counselling done at the sessions.

	State			Total (n = 136) n (%)
	Bihar (N = 62) n (%)	Gujarat (N = 43) n (%)	Kerala (N = 31) n (%)	
A. Beneficiaries vaccinated*				
A.1 Children vaccinated	748 (84.7)	567 (64.9)	339 (71.2)	1654 (74.1)
A.3 Pregnant women vaccinated	161 (122)	191 (68.7)	33 (54.1)	385 (71.2)
*B. Counselling services*				
B.1 Explained before vaccinating	36 (58.1)	28 (65.1)	21 (67.7)	55 (40.4)
B.2 Informed minor adverse effects	48 (77.4)	34 (79.1)	24 (77.4)	106 (77.9)
B.3 Reminded about the next visit	42 (67.7)	31 (72.1)	28 (90.3)	101 (74.3)
B.4 Keep immunization card safely	42 (67.7)	31 (72.1)	28 (90.3)	101 (74.3)
B.5 Delivered all 4 key messages	35 (56.5)	25 (58.1)	20 (64.5)	80 (58.8)
B.6 Asked to wait for 30 minutes#	32 (51.6)	17 (39.5)	6 (19.4)	55 (40.4)
B.7 Provided paracetamol	48 (77.4)	40 (93)	28 (90.3)	116 (85.3)

Note:

∗ Proportion of vaccinated children and pregnant women vs. scheduled as per due list (scheduled children as per due list: Bihar-883; Gujarat-874; Kerala- 476 and scheduled pregnant women as per due list: Bihar-132; Gujarat-278; Kerala-61).

# After vaccination the beneficiaries were advised to wait at the session site for assessing acute adverse events.

## 4 Discussion

This study documented the conduct, vaccine logistics, vaccine handling and vaccination related practices across 27 districts in three states of India. Availability of adequate space and the essential items are necessary for good session conduct and service delivery. We documented that all the essential items were available at only about half of the session sites. Updated beneficiary due list was not available at one-fifth of the session sites. Limited availability of beneficiary due list were also reported from different states of India including Assam (43.5%) Gujarat (48%, 61.6%, and 79%) and West Bengal (88%) [6, 7, 8, 9, 10]. Participation of community mobilisers (ASHAs and AWWs) is critical for improving the beneficiary turnout. Participation of mobilisers in three-fourth of sessions in the study is comparable to another report from Gujarat, where at 83.3% sessions a mobiliser was observed [7]. Over 100% beneficiary turnout in Bihar and Kerala point towards a probable incomplete due list and tally sheet or arrival of unscheduled visitors for vaccination. Lower turnout for the sessions in Gujarat indicated need for better community mobilisation. Low turnout at sessions has been also reported from Odisha (children-49.8% and pregnant women-64.8%) [11]. The lower turnout may also indicate towards the operational gaps indicated as a reason for missing vaccination during concurrent monitoring [2]. Limited availability of the hub cutters and sharp waste containers may pose challenge for safe disposal of used syringes and sharps.

Availability of at least one vial of each vaccine was limited at many session, especially for BCG and HBV vaccines. The lower availability of BCG and HBV vaccines might be due to higher institutional delivery and administration of the ‘zero’ dose to the newborns before discharge, especially in Kerala. Report from Uttarakhand also reported non-availability of all vaccines at the outreach sessions (BCG-45.8%; Hepatitis B vaccine-54.1%; Diptheria-Pertusis-Tetanus-87.5%; oral Polio vaccine-75%; Measles-79.1%) [12]. Similarly, all the vaccines were available at 76.6–94.7% of the sessions in Gujarat [8, 10, 13]. BCG and measles vaccine-diluent mismatches were observed at 9% and 2.3% of sites respectively. Such situations may promote refusal to vaccinate or adverse events by reconstitution using wrong diluent. Instances of missing labels on the vaccine vials/diluents, unusable VVMs and frozen vaccines indicate need for stringent cold chain and vaccine handling practices and care while packing. Auto-disable syringes were available at almost all the sites, which was comparable to reports from Gujarat (95%) higher than that from Uttarakhand (79.1%) [11, 12]).

Handwashing before vaccination was observed at half of the sites, which was similar across all states. This was comparable to reports from Assam (65.2%) and better than Uttarakhand (14%) [7, 14]. Although the responses from beneficiaries were not documented, but no handwashing may increase rick of contamination of during vaccine handling and administration and thereby the risk of immunization error adverse events. Handling of freeze-sensitive vaccines during session was inappropriate at 12% of the sites, which fall under the open vial policy and are at risk of freezing when placed in contact with icepack. Administration of frozen antigens may increase risk of minor local reactions and vaccine failure. No report on this practice was found from India. Time of reconstitution for BCG and measles vaccines were not noted in 10% of the sessions. Variability in this practice from other parts of India were also reported including Gujarat (61.5%, 88.3%, 100%), Uttarakhand (71%) and Assam (56.5%) [7, 8, 13, 14]. Compliance to counselling with the four key messages was observed at little over half of the sessions. Counselling practices at sessions sites reported to vary widely across the country (range 26–75%); Uttarakhand (28%), Gujarat (26.3%, 38.3%, 46%), Assam (73.9%) and West Bengal (75%) [7, 8, 9, 10, 13, 14]. At less than half of the sites, beneficiaries were advised to wait for 30 minutes, which was comparable to the reports from Gujarat and Assam (30–44%) [7, 10, 13]. Appropriate handling of syringes and needles after vaccination was observed at 50.7–63.2% of the sessions, which was comparable to reports from Gujarat (48.3%–77%) and Assam (65.2%) [7, 8, 13]. There were variations in the practices related to vaccine handling and waste management at the session sites across the states. All these studies indicated operational gaps in the conduct, adherence to the guidelines and supervision and monitoring processes for outreach sessions [7, 8, 9, 11, 12, 13, 14].

The outreach sessions provide a community level platform for immunization program service delivery and contribute significantly to the coverage in both rural and urban areas of India. Its effective implementation is important to sustain and improve the immunization coverage and also the public confidence. There are few reports on performance of the outreach sessions from India. The available reports have geography, methodology and rigor challenges. VHND platform is being implemented as single window portal for integrated immunization, maternal, child health, illnesses and other programmatic service delivery at community level. But in reality immunization is the primary service delivery happening at most of the VHNDs [15]. The low beneficiary turnout across different parts of India warrants regular updating the beneficiary due list and effective mobilisation. The gaps in vaccine logistics, handling during and disposal of used (empty/partially used) vaccine vials after the session need attention. Delivery of all the four key messages and waiting for 30 minutes after vaccination are to be reemphasised. There are ongoing efforts to improve immunization coverage along with system strengthening with special focus on head-count and expansion of sessions targeting the missed and partially vaccinated beneficiaries under Mission Indradhanush [16].

The spread over 27 districts and multiple states with focus on vaccine handling were the strengths. We did not assess the knowledge of vaccinators, routes of administration and satisfaction of the clients, which may be considered as the limitations.

This study documented the variations in the session organisation, logistics, vaccine handling and communication with clients related to the outreach immunization services across 27 districts of three states in India. There were more or less similar gaps observed across these states and need focus on adequate supply of vaccine and related supplies, training and supportive supervision for adherence to appropriate vaccine handling practices and client communication. There is need for regular updating the beneficiary list to ensure mobilisation improving accuracy and quality of vaccine demand and coverage data. Improvements in supervision and monitoring with social accountability are needed for improving the session performance and beneficiary experience to further the vaccine coverage and confidence.

## Declarations

### Author contribution statement

Manoja Kumar Das: Conceived and designed the experiments; Performed the experiments; Analyzed and interpreted the data; Contributed reagents, materials, analysis tools or data; Wrote the paper.

Narendra Kumar Arora: Conceived and designed the experiments.

Thomas Mathew, Bhadresh Vyas: Performed the experiments.

Monica Sindhu, Abhishek Yadav: Analyzed and interpreted the data.

### Funding statement

This work was supported by the Bill and Melinda Gates Foundation (grant number OPP1041395).

### Competing interest statement

The authors declare no conflict of interest.

### Additional information

No additional information is available for this paper.
